# Remarks on the Cure of Consumption

**Published:** 1844-01

**Authors:** Wm. A. M’Dowell

**Affiliations:** Louisville, Ky.


Art. IV.—*Remarks on the Cure of Consumption.* By Wm. A. M'Dowell, M.D., of Louisville, Ky.

"Cure of Consumption," or some such title, has, in times past, been assumed by so many swindling empirics, as the title of books or essays, which are mere laudations of themselves and their nostrums—their plasters, pills, or drops—compositions known only to them and some German or Turkish magician, or recommendations of some kind of long horn, valvular tube, or other *respirator* to cure by inflating the lungs—all puffed up and patented to the sole use and benefit of the wonderful discoverer, that it is not at all wonderful that the medical profession, disgusted with such knavery, should have grown weary of wasting their time in reading works that appear under such titles.

To this circumstance, at least, I am disposed to ascribe a less degree of attention than I think is merited, to a work published by me a few months since entitled "A Demonstration of the Curability of Pulmonary Consumption," &c. This work is the result of 25 years experience, investigation and labor, in which is set forth fully, fairly, and without reserve, a mode of treatment by which at least a proportion of from one-half to two-thirds of such cases of tubercular consumption (taken as they ordinarily occur to the practising physician) may be cured. Whether well or ill written, the full detail of a course of treatment, which actually effects cures of a hitherto unmanageable disease, merits attention, come whence it may.

The curability of consumption no longer rests on the results of my individual experience. I have, since the publication of my essay, above referred to, received communications from physicians in various quarters, who have cured cases treated on the plan therein suggested. The subjoined summary of which, together with the summary of results in my own practice, I trust will at least suffice to satisfy my brethren of the profession, that it might be as well to give it a trial, as to continue to prosecute the mode or modes of treatment, which so uniformly, through a period of more than two thousand years, have proved so signally unsuccessful. Almost any new method might be as safely pursued, as old ones by which, through so long a period, nearly all cases have been lost

From the 1st of January, 1843, to January, 1844, I have treated 84 cases of consumption; of these 12 have died, and 36 have been cured. A large majority of the remainder under treatment, are either convalescent, or improving; affording a fair prospect of a greater proportion of recoveries than the above.

Of the above cases, 61 were of Kentucky; 7 from Indiana; 4 from Tennessee; 4 from Louisiana; 3 from Mississippi; 1 from Virginia; 1 from Pennsylvania; 2 from Massachusetts; and 1 from New York.

Cures of cases have been reported to me by the following gentlemen:—three by Dr. N. Field, of Jeffersonville, Ia.; two by Dr. Griffith, of Jeffersonville, Ia.; two by Dr. B. H. Hawkins, of Raleigh, Tenn.; one by Dr. J. J. Moorman, of White Sulphur Springs, Va.; one by Dr. Shuck, of Lebanon, Ky.; one by Dr. Jennings, of Baltimore, Md.

Dr. Morgan of Rockbridge county, Virginia, whom I sometimes met in practice whilst I resided in Virginia, and who has for nine or ten years past treated the disease on the general principles suggested in my essay on consumption, informed me in December, that he had cured quite a considerable number of cases, and shewed me some of the rescued persons. He promised me to report his cases for insertion in this communication, but they have not yet come to hand.

*January*, 1844.

---

[A list of the names, residences, and present condition of the eighty-four cases in the above summary, which Dr. McD. does not feel himself at liberty to publish, is left at the office of the Western Journal of Medicine for the inspection of physicians or other persons, whose interest in the subject may render them desirous of a more minute investigation of the matter.—Y.]

